# Effects of work-related factors on self-reported smoking among female workers in call centers: a cross-sectional study

**DOI:** 10.1186/s40557-019-0286-8

**Published:** 2019-02-12

**Authors:** Young Joon Yang, Young Hoon Moon, Sang Yoon Do, Chul Gab Lee, Han Soo Song

**Affiliations:** Department of Occupational and Environmental Medicine, School of Medicine, Chosun University, Gwangju, Republic of Korea

**Keywords:** Call centers, Workplace violence, Occupational stress, Smoking, women

## Abstract

**Objectives:**

This study conducted to investigate work-related factors in relation to smoking among women working in call centers in Gwangju, South Korea.

**Methods:**

From 56 call centers (7320 employees), we selected 10 and conducted a survey using a structured questionnaire by randomly selecting 10% of workers from each center. A total of 387 subjects participated in this survey We analyzed for 375 respondents, after excluding men and those with missing responses. We analyzed the relationships of work-related factors such as emotional labor, workplace violence, employment type, annual salary, working hours, employment period with smoking, using multiple logistic regression analysis. Emotional labor and workplace violence were measured using the Korean Emotional Labor Scale (K-ELS) and Korean Workplace Violence Scale (K-WVS).

**Results:**

The prevalence of current smoking among call center female workers was 13.6%. Univariate analysis showed that “Emotional disharmony and hurt”, “Experience of psychological and sexual violence from supervisors and co-workers” among items of K-ELS and K-WVS, working hours, annual salary correlated with smoking. After adjusting for emotional labor, workplace violence, employment type, annual salary, working hours, employment period, and age, only working hours show a significant association with smoking. Women who worked 40–49 h had 3.50 times (95% CI = 1.04–11.80) and worked more than 50 h had 8.68 times (95% CI = 1.89–39.78) greater odds of smoking as compared with women who worked less than 40 h.

**Conclusions:**

Smoking was associated with working hours among female workers in call center. However, emotional labor and workplace violence did not show significant relationships with smoking.

## Background

Smoking is the leading preventable cause of cancer, cardiovascular disease, and respiratory disease in Korea [1]. According to Korean health statistics, the male smoking rate declined from 1998 to 2007 but has been stagnant since 2008. However, female smoking rates have remained unchanged since 1998 [2, 3]. In 2016, national health statistics showed that the smoking prevalence in women was 6.4%. The female smoking rate in South Korea was the lowest of all OECD countries. However, self-report measures may underestimate female smoking rates, because smoking among women is regarded more negatively in South Korea. The actual female smoking rate in South Korea is estimated to be more than twice as high as has been reported [4, 5].

One study analyzing data from the Fifth Korean National Health and Nutrition Examination Survey (KNHNE) showed that smoking rates in women differ significantly depending on the type of occupation. There was no significant difference by occupation group in smoking rates among males. However, women showed a significant difference by occupation group, with 4.7% in the non-manual group, 6.9% in the manual group and 9.4% in the service group [6]. Another study showed that odds ratio of smoking for service and sales female workers was 2.26 times (95% CI 1.31–3.90) higher than reference occupation (managers, professionals) [7]. This suggests that occupation-based approaches and investigation for work-related factors are needed to reduce female smoking rates. However, there is a lack of research on occupational causes of female smoking. The purpose of this study was to investigate the work-related factors affecting smoking among call center female workers who have reported high smoking rate [8–10].

## Methods

### Research subjects and data collection

Call center is a centralized office used for receiving requests of customers by telephone. An inbound call center deals with enquiries from consumers. An outbound call center is operated for sales calls, proactive customer service, debt collection and market research. Recently, most of the call centers are outsourced. In July 2017, 10 call centers were selected from 56 call centers considering the size and type of company in Gwangju, a metropolitan city. Six call centers were selected from the workplaces with less than 300 employees and four call centers from the workplaces with 300 or more employees. Five inbound and five outbound call centers were selected respectively. We explained the purpose of the study and all the centers agreed to participate. Ten percent of the workers at each call centers were randomly extracted using the employee number and asked to participate in a survey. We administered a structured self-completed questionnaire for workers who agreed to participate in the survey. A total of 387 workers responded to the questionnaire, which accounted for 5.3% of the 7320 call center workers in Gwangju. A total of 375 responses were analyzed, excluding 12 workers who were male or who returned insufficiently completed questionnaires. This study was approved by Chosun University Hospital Institutional Review Board (IRB No. CHOSUN 2017–06-009).

## Research tools

### General and occupational characteristics

We collected data about sociodemographic characteristics, working conditions (employment type, working hours, annual salary), health behavior (smoking rate, hazardous drinking rate) with a self-administered questionnaire. According to the WHO criteria, the hazardous drinking was defined as the case where the average amount of drinking per day was 3 cups or more and drinking more than 2 days per week. The employment types were classified into formal and informal workers (temporary, fixed contract). Working hours were classified as less than 40 h a week, more than 40 h a week and less than 50 h a week, more than 50 h a week. The annual salary was classified as less than 21.4 million Korean won (KRW) (lower 60% annual salary of Korean workers in 2016, data released by Korean Economic Research Institute) and 21.4 million KRW or more. Smoking rates were classified as current smoking and non-smoking.

### Korean emotional labor scale and Korean workplace violence scale

To assess emotional labor and workplace violence as distinctive job stressors of call center workers, we used the Korean emotional labor and workplace violence questionnaire [11, 12]. First, the Korean Emotional Labor Scale (KELS) consists of 24 questions in five areas of emotional demands and regulation, problems in the customer service process, emotional disharmony and hurt, organizational surveillance and monitoring, and organizational support and protection system. It uses a 4-point Likert scale (1 = not at all, 2 = not quite, 3 = somewhat, 4 = very). The Korean workplace violence scale (KWVS) consists of 24 questions in four areas of psychological/sexual violence from customers, psychological/sexual violence in the workplace from supervisor or coworkers, psychological/physical violence from customers/the workplace, and organizational support system for violence. We used the following criteria when summing the total scores for a more appropriate analysis.

[Emotional labor]

1. Emotional demands and regulation (5): Sum 0–15: Good, 16–20: Risk
2. Problems in the customer service process (3): Sum 0–9: Good, 10–12 Risk
3. Emotional disharmony and hurt (6): Sum 0–18: Good, 19–24: Risk
4. Organizational surveillance and monitoring (3): Sum 0–9: Good, 10–12: Risk
5. Organizational support and protection system (7): Sum 0–21: Good, 22–28: Risk

[Workplace violence]

1. Psychological/sexual violence from customers (4): Sum 0–8: Good, 9–16: Risk
2. Psychological/sexual violence in the workplace from supervisor or coworkers (4): Sum 0–4: Good, 5–16: Risk
3. Psychological/physical violence from customers/the workplace (2): Sum 0–2: Good, 3–8: Risk
4. Organizational support system for violence (14): Sum 0–42: Good, 43–56: Risk

### Data analysis

Chi-square tests was performed to investigate the differences in smoking rates according to potential factors such as age, working conditions, emotional labor, and workplace violence. A multivariate logistic regression analysis was utilized in order to calculate adjusted odd ratios of smoking for K-ELS and K-WVS items, employment type, annual salary, working hours, employment period. The significance level was set at *p* < 0.05 and SPSS 22.0 (IBM, New York, NY, USA) was used for the analyses.

## Results

### 1. General characteristics, working conditions, smoking rate, and risky drinking rate

Table 1 shows general characteristics of the subjects. Study participants were all women. Among the 375 total subjects, 225 (60.0%) were under 40 years and 150 (40.0%) were over 40 years of age. Regarding employment type, 64.5% were formal employees and 35.5% were informal employees. Regarding working hours, 93 subjects (24.8%) worked less than 40 h a week, 261 subjects (69.6%) worked more than 40 h and less than 50 h a week, 21 subjects (5.6%) worked more than 50 h a week. Regarding salary, 63.5% of subjects received less than 21.1 million won. Regarding employment period, 46.2% had worked less than 5 years. The rate of current smoking was 13.6% of subjects. The rate of risky drinking was 45.3%.

**Table 1:** General characteristics

Variables	Frequency	Percent
Age		
< 40	225	60.0
≥ 40	150	40.0
Employment type		
Formal	242	64.5
Informal	133	35.5
Working hours, per week		
< 40	93	24.8
40–49	261	69.6
≥ 50	21	5.6
^a^Annual salary (million KRW)		
< 21.4	238	63.5
≥ 21.4	134	36.5
No answer	3	0.8
Employment period (years)		
< 5	172	46.2
≥ 5	203	53.8
Smoking status		
Current smoker	51	13.6
Non-smoker	324	86.4
Alcohol intake		
Low risk drinking	205	54.7
Risky drinking	170	45.3

^a^In 2016, lower 60% annual salary of Korean workers was 21.4 million Korean won (KRW)

### Smoking rate according to potential risk factors

Table 2 shows the relationship between work-related factors and smoking. First, analyzing the association with smoking among the items of the K-Emotional Labor Scale showed that KELS-3 (“Emotional disharmony and hurt”) was related to smoking. Second, in the category of KWVS, only KWVS-2 (“Experience of psychological and sexual violence from supervisors and co-workers”) was significantly associated with smoking. Additionally, in the category of working condition, working hours and annual salary were related to smoking.

**Table 2:** Chi-square analysis with each risk factor and smoking

Variables		Non-smoker		Smoker		*p*-value
		*n*	%	*n*	%	
Emotional labor						
^a^KELS-1	Good	58	89.2	7	10.8	0.464
	Risk	266	85.8	44	14.2	
^b^KELS-2	Good	165	89.7	19	10.3	0.069
	Risk	159	83.2	32	16.8	
^c^KELS-3	Good	187	90.3	20	9.7	0.014
	Risk	137	81.5	31	18.5	
^d^KELS-4	Good	239	88.5	31	11.5	0.055
	Risk	85	81	20	19.0	
^e^KELS-5	Good	304	87.1	45	12.9	0.144
	Risk	20	76.9	6	23.1	
Workplace violence						
^f^KWVS-1	Good	262	87.3	38	12.7	0.292
	Risk	62	82.7	13	17.3	
^g^KWVS-2	Good	281	88.4	37	11.6	0.009
	Risk	43	75.4	14	24.6	
^h^KWVS-3	Good	290	86.6	45	13.4	0.785
	Risk	34	85	6	15.0	
^i^KWVS-4	Good	261	87.3	38	12.7	0.318
	Risk	63	82.9	13	17.1	
Working condition						
Employment type	Formal	210	86.8	32	13.2	0.774
	Informal	114	85.7	19	14.3	
Working hours (hours/week)	< 40	89	95.7	4	4.3	0.003
	40–49	220	84.3	41	16.7	
	≥50	15	71.4	51	13.6	
Annual salary (million KRW)	< 21.4	58	95.1	3	4.9	0.039
	≥21.4	266	84.7	48	15.3	
Employment period (years)	< 5	149	86.6	23	13.4	0.906
	≥5	175	86.2	28	13.8	
Demographic characteristics						
Age (years)	< 40	311	86.6	48	13.4	0.539
	≥40	13	81.3	3	18.8	

*p*-value by Chi-square test

^a^KELS-1: Emotional demands and regulation, ^b^KELS-2: Problems in the customer service process, ^c^KELS-3: Emotional disharmony and hurt, ^d^KELS-4: Organizational surveillance and monitoring, ^e^KELS-5: Organizational support and protection system, ^f^KWVS-1: Psychological/sexual violence from customers, ^g^KWVS-2: Psychological/sexual violence in the workplace from supervisor or coworkers, ^h^KWVS-3: Psychological/Physical violence from customers/the workplace, ^i^KWVS-4: Organizational support system for violence

### Related factors and their relationship with smoking

Table 3 shows odds ratios for smoking according to work-related variables such as emotional labor, workplace violence, work condition and age. Univariate analysis by χ2-test shows that KELS-3, KWVS-2, working hours, annual salary were related to smoking. However, after adjusting by each items of emotional labor and working violence, working condition such as employment type, annual salary, employment period, and age, only working hours was related to smoking. The adjusted odds ratio of smoking in the at-risk group for “emotional disharmony and hurt” was 1.77 times (95% CI 0.82–3.80) higher than that of the comparison group. The adjusted odds ratio of smoking in the at-risk group for mental/sexual violence in the workplace was 2.21 times (95% CI 0.94–5.21) higher than the reference group. For working conditions, in the group that worked more than 40 h, less than 50 h and more than 50 h, the adjusted odds ratios of smoking were 3.50 times (95% CI 1.04–11.80), 8.68 times (95% CI 1.89–39.78) higher than the group that worked 40 h or less.

**Table 3:** Odd ratios for smoking among call center female workers according to work-related characteristics

Variables	Unadjusted OR	95% CI	^a^Adjusted OR	95% CI
Emotional labor				
KELS-1, risk	1.37	0.59–3.20	0.87	0.34–2.23
KELS-2, risk	1.75	0.95–3.21	1.06	0.49–2.23
KELS-3, risk	2.12	1.16–3.87	1.77	0.82–3.80
KELS-4, risk	1.81	0.98–3.35	1.28	0.61–2.65
KELS-5, risk	2.03	0.77–5.32	1.19	0.40–3.55
Workplace violence				
KWVS-1, risk	1.45	0.73–2.88	1.04	0.47–2.31
KWVS-2, risk	2.47	1.24–4.95	2.21	0.94–5.21
KWVS-3, risk	1.14	0.45–2.86	0.53	0.17–1.60
KWVS-4, risk	1.42	0.71–2.82	0.98	0.45–2.13
Work conditions				
Employment type, Informal	1.09	0.59–2.02	0.85	0.42–1.69
Working hours,				
< 40 (hours/week)	1		1	
40–49 (hours/week)	4.15	1.44–11.92	3.50	1.04–11.80
≥ 50 (hours/week)	8.90	2.24–35.32	8.68	1.89–39.78
Annual salary, ≥21.4 million (KRW)	3.49	1.05–11.59	1.63	0.41–6.49
Employment period, ≥5 (years)	1.04	0.57–1.88	0.74	0.39–1.42
Demographic characteristics				
Age, ≥40(years)	1.5	0.41–5.44	2.48	0.56–10.96

^a^Adjusted by each items of emotional labor and working violence, working condition such as employment type, annual salary, employment period, and age

## Discussion

According to the results of this study, the smoking rate of female call center workers was 13.6%. Research on the prevalence of smoking among female workers in call center is rare. One study that conducted a smoking cessation program among 301 female workers in a call center reported that the smoking rate was 15.9% [9]. A study of the hearing thresholds of call center workers found that women smoke rate was 16.1% [13]. In the 2012 Seoul Women’s Healthcare Project, a survey was conducted on 4939 women who randomly sampled their workplaces. A total of 716 call center workers were surveyed in this study, and the smoking rate was 26.0% [10]. We conducted an anonymity questionnaire that did not collect personal information and tried to reduce the under-reporting on smoking, but it is possible that the self-reported smoking rate was estimated to be lower than actual smoking.

The authors were interested some studies that psychosocial distress or job stress is associated with smoking [14–17]. Psychologic stress is known to be a factor that makes smoking cessation difficult [18]. Therefore, we tried to find out whether emotional labor and work violence, which are major stressors of call center workers, are related to smoking. Interestingly, emotional labor and workplace violence were not significantly associated with smoking in our study. This result may be due to the overall high level of emotional labor and workplace violence among female workers in the call center. For workplace violence, violence from customers was not relevant (adjusted odds ratio 1.04, 95% CI = 0.47–2.31), but experiencing psychological and sexual violence from supervisors and co-workers in the workplace show relatively higher adjusted odds ratio (2.21, 95% CI = 0.94–5.21). The authors guessed that emotional labor and customer violence are predictable and adjustable stresses, but violence in the workplace is unpredictable or more stressful.

In this study, longer working hours were significantly related to higher smoking rates. Cho et al. reported that smoking rate in women was 5.5% in the group working < 40 h a week, 6.8% in 40–48 h, 12.3% in 49–60 h, 5.2 in > 60 h [6]. Furthermore, a study using the 3rd KNHNE showed dose-response relationship between working hours and smoking in women [19]. The results of this study support previous findings that smoking was related to long working hours even in women. The higher the household income or socioeconomic level, the lower the smoking rate is reported [20, 21]. Nevertheless, one study using the third Korean Working Condition Survey showed that smoking rates tend to be higher with higher levels of annual salary in women [13]. Furthermore, another study using fifth KNHNE showed that while the rate of smokers was lower for males as household income increased, females did not show a significant relationship with household income and smoking rates [6]. Our study did not showed significantly relationship between smoking and annual salary among women workers in call center. Perhaps there are various interactions when annual salary levels or socio-economic levels affect smoking.

In our study, age was not significantly related to smoking. However, in general, smoking rates tend to decrease with age. As age increases, there is more awareness about health and smoking cessation, considering the risk of secondhand smoke in the family. However, this trend may not have appeared in this study. In this regard, a qualitative study noted that smoking was continued as providing a smoking room nearby where workers can smoke freely and using smoking as a means of improving work efficiency in call centers [8].

This study has the following limitations. First, Self-reported smoking rates may be lower than actual smoking rates. Although the anonymous survey was carried out, there may be a fear of social stigma. Second, experience of smoking before entering the company, the presence of smokers in the family, and the smoking status of the parents were important factors, yet this study did not investigate these variables. Third, the company’s smoking cessation policy is a very important factor in reducing the smoking rate [22]. However, it was not investigated as a variable in this study. Finally, because this study is cross-sectional, causality cannot be explained solely by the significant factors shown in this study.

## Conclusion

According to the results of this study, long working hours was an important factor in the smoking rate of female workers at the call center. On the other hand, emotional labor and work violence which were expected to affect smoking as stress factors, were not significantly related. This result may be due to the overall high level of emotional labor and workplace violence in female workers at the call center. Therefore, further studies including other occupations are needed to understand the effects of emotional labor and workplace violence on smoking.

## Abbreviations

KELS: Korean emotional labor scale
KNHNE: Korean national health and nutrition examination survey
KWVS: Korean workplace violence scale
KRW: Korean won
